# ADHD remote technology and ADHD transition: predicting and preventing negative outcomes (ART-transition) - an adolescent prospective cohort study protocol

**DOI:** 10.1186/s12888-025-07546-0

**Published:** 2025-12-11

**Authors:** Aislinn Bowler, Johnny Downs, Ewan Carr, Madeleine Groom, Andrea Bilbow, Anna Redly, Katie Cunningham-Rowe, Amos Folarin, Richard J. B. Dobson, Jonna Kuntsi

**Affiliations:** 1 Social, Genetic and Developmental Psychiatry Centre, Institute of Psychiatry, Psychology and Neuroscience, King’s College London, London, UK; 2 School of Academic Psychiatry, Institute of Psychiatry, Psychology & Neuroscience, King’s College London, London, UK; 3 The Department of Biostatistics and Health Informatics, Institute of Psychiatry, Psychology and Neuroscience, King’s College London, London, UK; 4 School of Medicine, Mental Health & Clinical Neurosciences, University of Nottingham, Institute of Mental Health, Nottingham, UK; 5 NIHR MindTech MedTech Health Research Centre, Institute of Mental Health, University of Nottingham, Nottingham, UK; 6 ADDISS, The National Attention Deficit Disorder Information and Support Service, Edgware, Middlesex UK; 7 Institute of Health Informatics, University College London, London, UK; 8 NIHR Biomedical Research Centre at South London and Maudsley NHS Foundation Trust and King’s College London, London, UK; 9 Health Data Research UK, University College London, London, UK; 10 NIHR Biomedical Research Centre at University College London Hospitals NHS Foundation Trust, London, UK

**Keywords:** Attention deficit hyperactivity disorder, ADHD, Mobile-health, Mental health, Adolescence, Remote monitoring

## Abstract

**Background:**

Late adolescence is a highly challenging and potentially critical period for young people with attention deficit hyperactivity disorder (ADHD). Many of the conditions that frequently co-occur with ADHD, such as depression, often first emerge in adolescence. Major life transitions, such as moving out of the parental home, lead to multiple new demands and changes in available support networks, further increasing the vulnerability. Additionally, UK data show that most young people with ADHD do not successfully transfer to adult services and thus do not receive appropriate interventions at a time when they may need them most. The lack of well-defined targets for holistic interventions limits opportunities for intervention. Our project on ADHD transition - ADHD Remote Technology and ADHD Transition: predicting and preventing negative outcomes (ART-transition) - uses remote measurement technology (RMT), which offers the potential to obtain ongoing, long-term, real-world data. We aim to identify the nature and timing of real-world changes in the transition to adulthood and what predicts such changes. We will further use these timeframes and intervention targets to co-design, with young people with ADHD, a prototype for an ADHD transition interactive smartphone app.

**Methods:**

ART-transition is a prospective cohort study that will recruit 250 16-17-year-olds with ADHD. The participants will complete baseline assessments, including an ADHD clinical interview, questionnaires, and cognitive tasks. Participants will then be monitored remotely over 24 months. Passive monitoring, which involves the participants wearing a wrist-worn device (Fitbit Charge 6) and downloading the study Passive App on their smartphone, will provide ongoing data collection on a range of variables, such as physical activity, sleep, heart rate, smartphone usage including social connectivity, and the environment (e.g. ambient noise, relative location). Active remote monitoring involves the participant downloading the study Active App to complete tasks (such as clinical questionnaires and speech tasks) and using a PC/laptop to complete cognitive tasks. The ADHD Remote Technology (ART) system is built on the RADAR-base mobile-health platform.

**Discussion:**

ART-transition will use RMT to identify fluctuations in ADHD symptoms and the wider phenotype at a level of detail not previously possible and to identify real-world targets for intervention.

**Clinical trial number:**

Not applicable.

**Supplementary Information:**

The online version contains supplementary material available at 10.1186/s12888-025-07546-0.

## Background

Late adolescence and the transition to adulthood is a highly challenging period for young people with attention deficit hyperactivity disorder (ADHD) that can lay the foundations for diverging adulthood trajectories. Many of the conditions that frequently co-occur with ADHD, such as depression, delinquency and substance misuse, often emerge in adolescence [1–3]. Major life transitions at this age, such as leaving education, starting work or moving out of the parental home, lead to multiple new demands and changes in available support networks, further increasing the vulnerability of young people with ADHD. This vulnerable phase coincides with the clinical transition from child and adolescent mental health care to adult services, which itself is a focus of major current clinical concern: UK data show that a large proportion of young people with ADHD do not successfully transfer to adult services, despite significant needs for ongoing treatment [4].

While people with ADHD are, at a group level, at an increased risk for outcomes such as educational underachievement, unemployment, and the development of many co-occurring psychiatric and physical disorders [5], individual long-term trajectories can be highly variable. Evidence for such variability in individual trajectories is emerging both for the development of comorbid conditions [6, 7] and the severity of ADHD symptoms and impairment [8]. Data from the Multimodal Treatment of ADHD study (MTA) challenge past notions of long-term ADHD trajectories by showing that for most of the sample (64%), the follow-up period from childhood to young adulthood was characterised by *fluctuating* ADHD persistence and remission; only 11% demonstrated stable persistence across all time points, and 9% sustained remission [8]. These data suggest that ADHD, though linked to a biological vulnerability, is a condition that may fluctuate over time in symptoms and impairment more than previously acknowledged, likely in response to environmental factors or health behaviours. The identification of such modifiable factors, which may serve as targets for new environmental or health interventions, emerges as a research priority. Overall, we lack detailed data on the extent to which ADHD and co-occurring symptoms fluctuate in late adolescence, what predicts improvement, worsening, or the emergence of new symptoms, and how such changes in clinical symptoms and functioning relate to other changes occurring during this period.

Past research has identified only limited clinical, cognitive and socio-economic predictors of the persistence of ADHD symptoms and functional impairment over time, such as ADHD severity, presence of co-occurring psychiatric symptoms (e.g. depression, oppositional problems), lower general cognitive ability, low SES and male gender [9–12]. The narrow range of predictors assessed and the long assessment intervals of several years mean that such data are of limited use for understanding the changes taking place during the ADHD transition phase. Potentially promising data on modifiable lifestyle factors has come from research on physical activity [13–19]. For example, in a longitudinal population-based study of 232 identical twin pairs, greater energy expenditure at age 16–17 predicted reduced ADHD symptoms at age 19–20, even after adjusting for unmeasured confounding using a within-identical twins design [16]. The evidence from RCTs for ADHD medication as a predictor of improvement in clinical symptoms in the short term is strong [20], but we need data on a wider range of longer-term outcomes during ADHD transition. Most acutely, treatment non-adherence is exceedingly common during ADHD transition, yet is poorly understood [17]. Data from UK primary care suggest only 18% of young people prescribed medication for ADHD in their early teens continued to receive prescriptions beyond the age of 18 [21]. Even when receiving prescriptions, individuals often do not take their medication regularly. We need to better understand the reasons for treatment non-adherence among young people with ADHD and establish if treatment adherence, jointly with lifestyle factors such as physical activity, predicts optimal functioning. The available limited data suggest that the presence of some comorbidities, such as affective disorders and autism, may increase the likelihood of continued treatment during transition; yet other data link autism to a more difficult transition [22].

Remote measurement technology (RMT) offers unprecedented opportunities for addressing current research and clinical challenges. For longitudinal research on ADHD, RMT enables us to move from infrequent ‘snapshot’ assessments in artificial lab or clinic settings on modest sample sizes, to frequent or ongoing, long-term, real-world data collection on a wide range of novel as well as conventional measures, in large, geographically representative samples. For the current project on ADHD transition, RMT offers the potential to both identify fluctuations in symptoms and the wider phenotype at a level of detail not previously possible, and to identify real-world targets for intervention that include environmental factors and health behaviours. The project benefits from our ADHD Remote Technology (ART) remote monitoring system for adolescents (ages 16+) and adults with ADHD, which we have previously successfully applied in our ART pilot study [23–26] and our ongoing ‘ART-CARMA’ project on adults with ADHD (ADHD Remote Technology study of cardiometabolic risk factors and medication adherence [27]). ART consists of both active (questionnaires, cognitive tasks, speech task) and passive (e.g. physical activity, sleep, smartphone use and sensor data) monitoring using mobile and web technologies. The measures are collected via the RADAR-base mobile-health platform [28], previously applied to other disorders, such as depression [29]. The ART pilot study provided support for the acceptability, feasibility and validity of the approach and measures [23–26]. Using a within-individual design allows us to control for many potential confounders [30], while regularised multivariable prediction models will allow us to capture complex temporal relationships between a range of measures and ADHD outcomes.

ADHD is diagnosed, through clinical interview, when an individual exceeds a threshold for symptoms of inattention and/or hyperactivity-impulsivity, and if the symptoms cause significant functional impairment. A challenge during the transition phase to adulthood is that the main informant for ADHD diagnostic symptoms changes from parent (and teacher) to self; yet data show that agreement between parents and young people with ADHD is only modest [31]. While self-report of symptoms and functional impairment has, necessarily, validity for clinical decision-making, the limited agreement between parents and young people with ADHD highlights the importance of extending the measurement of symptoms and impairments to objective digital and cognitive markers, which can further contribute to an improved understanding of the underlying processes.

Promising digital signals for other disorders have emerged from studies such as Remote Assessment of Disease and Relapse – Major Depressive Disorder (RADAR-MDD), where worsening depression was linked, for example, to over-time changes in speech collected using a study Active App [32] and a reduced nearby Bluetooth device count as a proxy for social isolation [33]. The latter emerged from the RADAR-base Passive App, which collects ongoing data from the smartphone sensors on novel digital markers ranging from smartphone-use behaviours (e.g. sociability, app usage) to background data such as relative location, ambient noise and light. In the ART pilot study, using Active and Passive App data collected from 40 participants with and without ADHD over a 10-week remote monitoring period, we identified candidate ADHD digital signals of restlessness, inconsistent attention, and difficulties completing tasks [23]. While cognitive tasks allow well-established measurement of cognitive differences associated with ADHD, past longitudinal studies are limited to a few (often just two) time points [7, 34–38]: we do not know whether changes in cognitive functioning are stable or fluctuate over time. In ART-pilot we developed and validated self-administration versions of two ADHD-sensitive cognitive tasks, enabling repeated assessment over time [24]. Our past studies further suggest a link between attention dysregulation and arousal dysregulation in people with ADHD [39, 40]. Wearable technology now enables us to move the measurement of, for example, heart rate from the lab to the real world. Overall, the long-term, ongoing RMT real-world data collection in the current project provides an opportunity to assess a wide range of detailed candidate objective markers of clinical symptom severity, in addition to the Active App being used to obtain self-ratings and the web-based Research Electronic Data Capture (REDCap) web-based platform to obtain informant-ratings on clinical questionnaires.

The discovery of genome-wide significant risk loci for ADHD enables us to derive a polygenic score (PGS) for each individual that captures the genetic signal underlying ADHD [41]. ADHD PRS is associated with many co-occurring psychiatric traits [42, 43], as well as with attention regulation in people with ADHD measured with tasks comparable to those applied in the present study [38]. We will thus account for ADHD genetic risk in ART-transition by incorporating an ADHD PGS. Capturing its contribution even in more modestly sized samples will bring added value.

### Participant involvement in the ART‑transition study protocol

The ART system benefits from feedback from participants in the ART-pilot feasibility study on the acceptability of the chosen measures and procedures [25]. Of the participants in ART-pilot, 95% agreed that there is value in gathering data through RMT, with some participants explaining that passive monitoring provides a unique opportunity to collect objective data. All participants agreed that the study measures blended into their daily lives, and 82% of participants expressed positive views about using the system for at least one year. The ART-transition study further benefits from the service user focus group for the ADHD Remote Technology study of cardiometabolic risk factors and medication adherence (ART-CARMA) study, which uses the ART system in adults with ADHD. The feedback from the ART-CARMA focus groups informed this study design regarding the frequency of questionnaire administration and the acceptability of measures.

In preparation for using the ART system with young people (aged 16+), we conducted several focus groups with young people. The groups included a Young Persons’ Mental Health Advisory Group session (*n* = 8), young people with ADHD who had recently transitioned from child and adolescent mental health services (CAMHS) to adult ADHD services (*n* = 10), and two young people with ADHD and a parent of a young person with ADHD (*N* = 3). In these groups, participants described the transition as a difficult process. They also viewed the development of an ADHD transition app positively and made suggestions on potential components. We also gained feedback on adapting our “About You” demographics questionnaire for young people, an ADHD symptom daily symptom question, and the participant and summary information sheets. Feedback from all the focus group sessions has been incorporated into the study design. Lastly, we have set up a patient and public involvement (PPI) panel (*N* = 7) to provide feedback throughout the study on areas such as recruitment, debrief interviews, participant feedback, and interpreting the results.

### Study objectives

This project, ADHD Remote Technology and ADHD transition: predicting and preventing negative outcomes (ART-transition), will use RMT in adolescents with ADHD to collect unobtrusive, real-world data over 24 months. By recruiting 250 16-17-year-olds with ADHD and monitoring them remotely, we can address the following three main aims: Aim 1) To identify, with precision, the nature and timing of the real-world changes that take place in the transition to adulthood; Aim 2) To identify what predicts such changes; and Aim 3) To use the timeframes and intervention targets identified in Aims 1–2 to co-design, with young people with ADHD, a prototype for a new ADHD-transition interactive smartphone app.

Aim 1 incorporates five objectives: To identify changes in (1a) ADHD symptoms and functional impairment; (1b) new co-occurring psychiatric symptoms that may emerge (e.g. anxiety, depression, irritability, substance use, antisocial behaviour); (1c) treatment adherence and engagement with clinical services; and (1d) healthy lifestyle behaviours (e.g. physical activity, sleep, daily structure, online lifestyle), social support and employment/ studies; and (1e) To identify objective markers – digital signals and cognitive measures – that are associated with changes in the clinical symptoms. Aim 2 focuses on the development and validation of multivariable prediction models for each of the changes (outcomes) identified in Aim 1, incorporating four objectives: To identify factors that predict changes in (2a) ADHD symptoms and functional impairment; (2b) co-occurring symptoms; (2c) treatment adherence and engagement with clinical services; and (2d) adoption of healthy lifestyle behaviours and engagement with work/studies. Information on influential predictors and their timing will feed directly into Aim 3 of developing a prototype ADHD-transition smartphone app.

## Method

### Study design

ART-transition is a prospective, observational, non-randomised, non-interventional study that uses wearable technology and smartphone sensors to remotely monitor 250 16–17-year-olds with ADHD for 24 months. The study does not represent any change to participants’ usual care or treatments due to participation. There is no control comparison group or randomised group allocation.

### Study population

The sample sizes were determined by simulation and analytical methods. For Aim 1, we considered power to detect associations between pairs of variables (e.g., ADHD symptoms and digital markers), provide precise of estimates of within-person variance and intraclass correlation (ICC), and the impact of missing data. For Aim 2, we estimated the sample size needed to develop multivariable prediction models (based on an expected model performance of R² ≈ 0.5 and 20 model parameters). Full details are provided in the Supplementary Materials. Overall, a sample of 250 participants (each providing monthly outcome assessments and continuous RMT data collection) will be sufficient to detect associations and estimate within-person variance with precision (Aim 1), even under conservative assumptions about missing data. This sample will be adequate to develop multivariable prediction models. Table 1 summarises the study eligibility criteria.

**Table 1:** Eligibility criteria for participation in ART-transition

Participant inclusion criteria	1	Diagnosis of DSM-5 ADHD
	2	Aged 16–17
	3	Able to give informed consent for participation
	4	Willing and able to complete self-reported assessments via smartphone
	5	Willing to use either their own compatible Android phone or a study Android phone as their only smartphone during the data collection period
	6	Willing to wear the wearable device (Fitbit) during the data collection period
Participant exclusion criteria	1	Psychosis, currently experiencing a major depressive episode, mania, drug dependence in the last six months, or a major neurological disorder
	2	Recent contact with psychiatric acute care (admission, crisis team or liaison team (A&E)) in the last six months
	3	Any other major medical disease which might impact upon the patient’s ability to participate in normal daily activities (e.g., due to hospitalisations)
	4	Pregnancy
	5	IQ < 70
Informant inclusion criteria	1	A parent or guardian, as chosen by the participant with ADHD
	2	Aged 18 or over
	3	Willing and able to complete web-based questionnaires regarding the participant with ADHD

### Study procedures

**Fig. 1:**
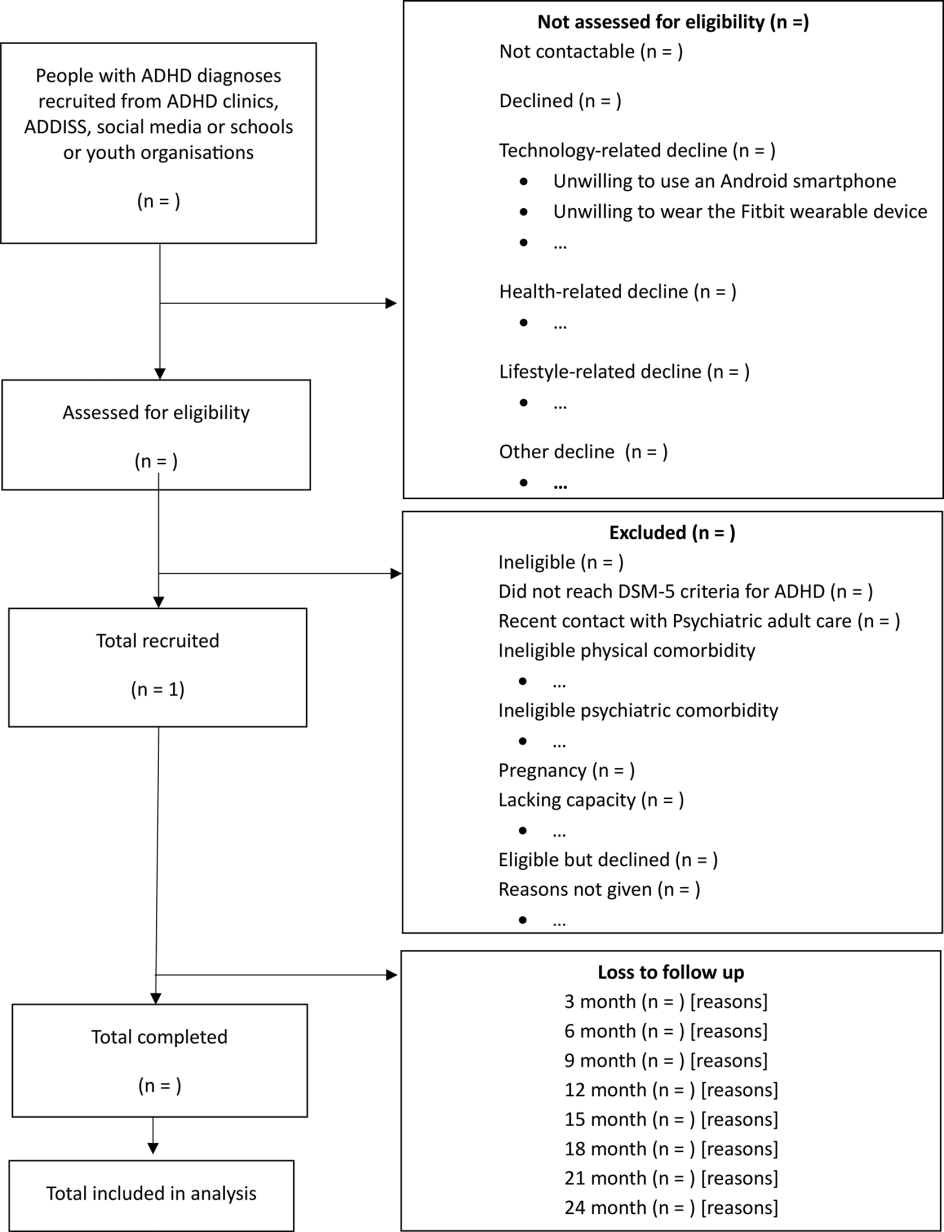
Participant flowchart

We will recruit 250 young people from CAMHS and private ADHD clinic lists, from the Consent for Contact (C4C) NHS Clinical Record Interactive Search (CRIS) database of patients [44], social media, the ADHD information service charity ADDISS, and through schools and youth clubs. The flow of participants through the study, including reasons for not participating in the study, will be documented in a flowchart (Fig. 1). The schedule of events is available in Table 2.

**Table 2:** Schedule of events for ART-transition

Month	0		1	2	3	4	5	6	7	…	24
Assessment	1	2									
Baseline assessments											
Study explanation^a^	X										
Informed consent^a^	X										
Introductory training^a^		X									
ADHD diagnostic interview (DIVA-5)^a^	X										
IQ and short-term memory WASI-II and WAIS-IV^a^	X										
Cognitive tasks (CPT/GNG and fast track)^a^		X									
‘About You’: Socio-demographics, medical history, service use, experience with Technology, life experiences^a,b^	X										
Autism Spectrum Disorder (AQ-10)^a,b^	X										
DNA saliva sample^a^		X									
Remote data collection											
Fitbit Charge HR6: Physical activity, sleep, heart rate, breathing rate and skin temperature^c^			Continuous (months 1–24)								
Passive App (smartphone sensors: relative location, light, smartphone use, battery life)^c^			Continuous (months 1–24)								
Medication use^d^			Daily (months 1–24)								
ADHD symptoms^d^			Daily (months 1–24)								
Medication side effects (CADDRA)^d, e^			XXXX	XXXX	XXXX	XXXX	XXXX	XXXX	XXXX	XXXX	XXXX
ADHD (BAARS-IV and functional impairment scale-self)^d^		X	X	X	X	X	X	X	X	X	X
Anxiety (GAD-7)^d^		X	X	X	X	X	X	X	X	X	X
Depression (PHQ-8)^d^		X	X	X	X	X	X	X	X	X	X
Aggression (RPQ-A)^d^		X	X	X	X	X	X	X	X	X	X
Irritability (ARI - self)^d^		X	X	X	X	X	X	X	X	X	X
Smoking (Test Fagerstrom)^d^		X	X	X	X	X	X	X	X	X	X
Alcohol use (AUDIT)^d^		X	X	X	X	X	X	X	X	X	X
‘About You’ – remote version^d^			X	X	X	X	X	X	X	X	X
Speech sample task^d^		X	X	X	X	X	X	X	X	X	X
ADHD (BAARS-IV and functional impairment scale–informant report)^b^		X	X	X	X	X	X	X	X	X	X
Irritability (ARI – informant report)^b^		X	X	X	X	X	X	X	X	X	X
Cognitive tasks (CPT/GNG and fast task)		X						X			X
Self-esteem (Rosenberg self-esteem scale)^d^		X						X			X
Eating behaviours (EDE-QS)^d^		X						X			X

^a^With researcher

^b^REDCap web-based platform

^c^RADAR-base Passive App

^d^RADAR-base Active App

^e^Delivered four times per month (every week)

Recruitment through clinical and private CAMHS teams involves the clinical teams contacting relevant individuals by their preferred method of contact (post, email, or telephone) and providing them with information about the study. If they consent for their details to be passed onto the research team, the research team will contact the patient and provide them with further information. If the patient is interested in participating, the first baseline assessment with the research team will be scheduled.

The baseline assessments take approximately four hours to complete across two days, with approximately a week between sessions. The virtual baseline sessions consist of (1) the Diagnostic Interview for ADHD in Adults (DIVA [45] to have a record of current ADHD symptoms and clinical impairment (2), a brief IQ and short-term working memory test [46, 47] (3) two baseline questionnaires: a demographic About You questionnaire and the Autism Spectrum Quotient questionnaire (AQ-10 [48]), (4) two cognitive tasks of the Combined Cued Continuous Performance Test and Go/NoGo (CPT/GNG) task and the Fast Task (while researcher present) [24], (5) completion of questionnaires (see Table 2) (6), a training session on the smartphone and wearable device, and (7) guidance on how to collect DNA saliva sample (optional). Participants are able to take breaks during the sessions if required. During the baseline sessions, the participants are also asked to identify a partner, a family member or a close friend who could complete informant report versions of the active monitoring questionnaires on ADHD symptoms, impairment, and irritability. The participants are asked to forward an email to their chosen informant, which asks the informant to get in touch with the research team if they are happy to participate. If the informant consents to participation, they will be sent online questionnaires using the REDCap web-based platform.

### Remote data collection

**Fig. 2:**
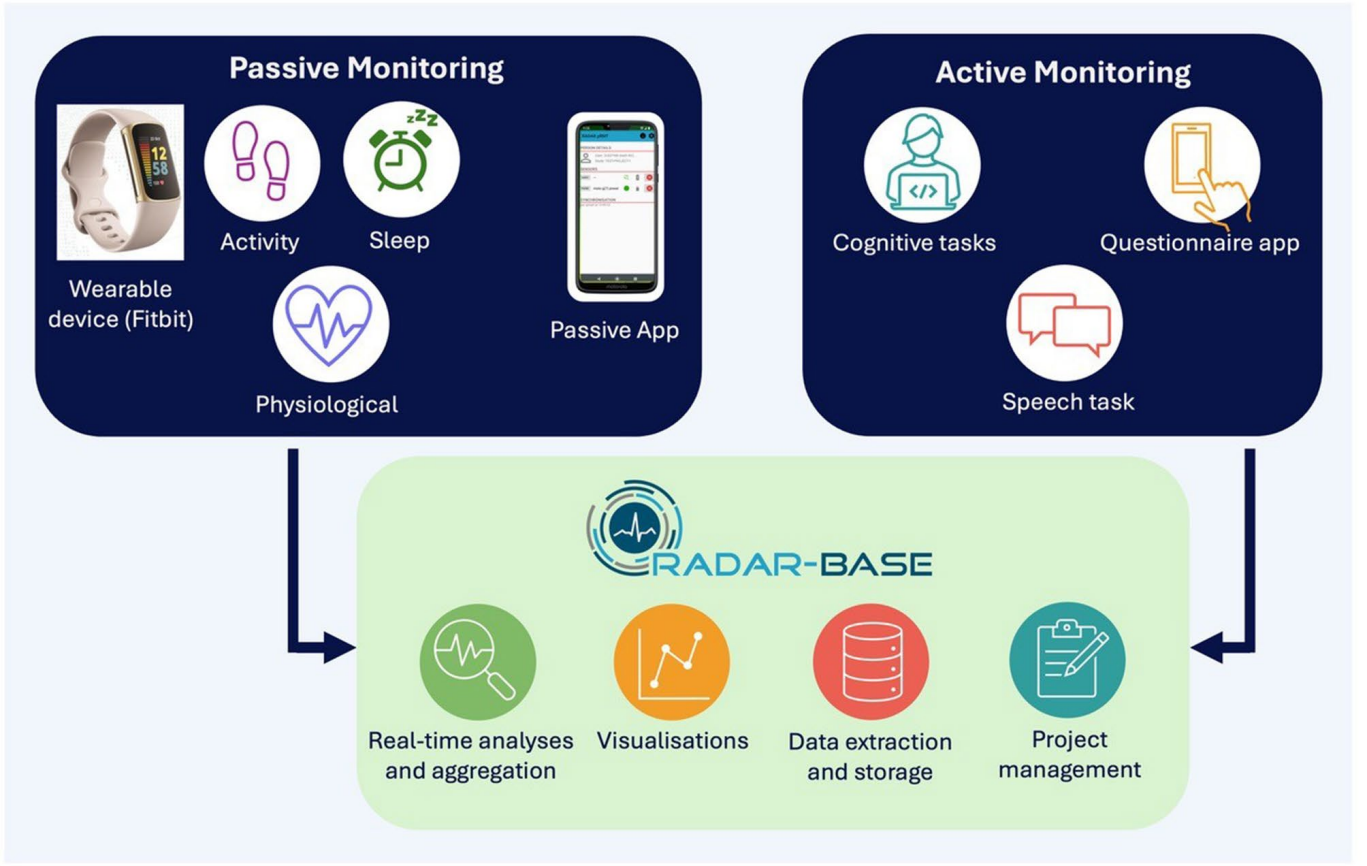
Remote monitoring system for ART-transition: passive and active data flows

Remote monitoring begins immediately after the two baseline assessments and lasts for 24 months. The remote monitoring consists of both active and passive components, following procedures established for the wider ART research programme, and is linked to the RADAR-base mobile-health platform [28]. Below is a summary of the methods of remote data collection used in the ART-transition study (Fig. 2).

#### Compatible android smartphone

The RADAR-base Passive App requires the use of an Android phone running the Android 10 operating system or newer. Participants without a suitable smartphone are provided with a compatible Android device. If this requirement creates a significant barrier to recruitment, we will consider relaxing the inclusion criteria of “*Willing to use either their own compatible Android phone or a study Android phone as their only smartphone during the data collection period”* to allow a subset of participants to retain their iPhone if they do not wish to switch to an Android phone. These participants will thus not download the Passive App.

#### Active app

Participants are asked to download the RADAR-base Active App, which asks the participant to complete questionnaires at specific intervals. Participants are asked to complete two short questionnaires every day. The first is a single-item questionnaire assessing the impact of their ADHD symptoms on that day, rated on a scale of 1–5. The second is for medication use. If participants are on medication for ADHD, they are asked if they took their medication that day, with follow-on questions on the type and dose of medication or why medication was not taken (e.g., experienced side effects, forgot to take their medication, or chose not to take their ADHD medication that day). If participants indicate at baseline or in the remote monitoring period that they do not currently take medication, this questionnaire will be paused for one month before asking them again if they are currently taking ADHD medication.

Participants are asked to report their medication use side effects weekly using the CADDRA Patient ADHD Medication form [49]. This form contains four items on changes experienced since starting medication and 28 items on side effects. Every four weeks, participants are asked to complete a longer set of questionnaires: ADHD symptoms and impairments will be measured using the Barkley Adult ADHD Rating scale on current symptoms (BAARS-IV) [50] and Barkley ADHD functional impairment scale [51]. This includes the 18 diagnostic ADHD symptoms and 10 items on functional impairment. Depression is measured using the 8-item Personal Health Questionnaire Depression Scale (PHQ-8 [52]), which is a widely used measure of current depression in the general population; anxiety is measured using the 7-item Generalized Anxiety Disorder questionnaire (GAD-7 [53]), which provides a dimensional score of anxiety symptoms; aggression is measured using the 23-item Reactive-Proactive Aggression Questionnaire for Adults (RPQ-A [54]); irritability is measured using the self-reported 7-item Affective Reactivity Index questionnaire (ARI-s [55]); smoking behaviours are measured using the 6-item Test Fagerstrom questionnaire [56]; and alcohol use is measured using the Alcohol Use Disorders Identification Test (AUDIT [57]). In addition, participants are asked to complete a remote monitoring version of the “About you” questionnaire, which includes questions on changes to life circumstances, life events, medication use, and other ADHD-related and non-ADHD-related treatments. Every six months, participants are asked to complete two further questionnaires. Positive and negative feelings about the self are measured using the 10-item Rosenberg Self-Esteem Scale [58], and eating disorder symptoms are measured using the 12-item Eating Disorder Examination Questionnaire (EDE-QS) [59].

Participants are asked to complete a short speech task every four weeks on the Active App. They also self-administer the two cognitive tasks of CPT/GNG and Fast task four times during the 24 months (months 6, 12, 18, and 24) on their home PC or laptop, without the researcher present. In addition, ratings on ADHD symptoms are obtained monthly from a partner, a family member, or a close friend of the individual with ADHD using the web-based REDCap.

#### Passive app

Participants are asked to download the RADAR-base passive remote monitoring app, configured for ART requirements [32], which runs in the background, requiring no further input from participants. The Passive App collects data on ambient noise, ambient light, phone usage information (e.g., which apps were used and for how long, when the phone was unlocked), passive audio, Global Positioning System (GPS) location, Bluetooth connectivity, battery life, gyroscope data, steps, and acceleration. Features of the passive audio, rather than raw audio itself, are extracted from the audio on the phone for transmission. GPS location data are randomised; that is, providing relative location data, not absolute coordinates. This prevents the identification of an individual’s home address or precise geographical location.

#### Wearable sensors

The wearable device in ART-transition is a low-cost, widely available, wrist-worn fitness tracking device (Fitbit Charge 6). Participants are asked to wear the device for the duration of the study (24 months), which provides ongoing data collection of physical activity, sleep, heart rate, breathing rate and skin temperature. Device sensors include a gyroscope, accelerometer, thermometer and photoplethysmographic pulse oximeter. Intraday series include activity metrics (per 1 min), heart rate (per 1 s), SpO2 (per 1 min), and breathing rate (per 1 min). The activity tracking feature records step count, distance travelled, floors climbed, elevation and intensity minutes (Metabolic Equivalent of Task, MET). Sleep statistics include the duration of sleep, sleep levels, sleep efficiency, time to sleep, and time in bed after waking. Data are collected from the Fitbit representational state transfer application protocol interface (REST-API) using the RADAR-base platform.

### Follow‑up procedure and data monitoring

Participants are contacted by telephone one month after their baseline assessment to address any concerns or questions. Brief follow-up phone calls are then offered every three months to maintain engagement with participants throughout the course of the study and to remind participants of any upcoming assessments. Participants are encouraged to live their lives as normal, responding to the Active App notifications when required. The research team are able to review if data is coming through using the RADAR-base management portal. The research team may contact participants if there is a loss of data from a device.

### Study debrief

At the end of the follow-up period (24 months), participants will receive a ‘debrief’ session, which will serve to collect endpoint acceptability and usability outcomes and to retrieve study materials (devices, chargers, etc.). This will be done either over the phone or on Microsoft Teams with a member of the research team and will be audio recorded using Microsoft Teams. Participants will also have an opportunity to view and discuss summaries of their data.

### Adverse events and study withdrawal

There may be several reasons for withdrawal from the study:

1. Participant choosing to no longer participate: Participants will be informed both in writing and verbally that participation is voluntary, that non-participation will not influence their medical care, and that they are free to withdraw from the study at any point without providing reasons.
2. The research team may withdraw the participant in the event of intercurrent illness, adverse event (AE), protocol violation, administrative or other reasons.

In the event of participant self-withdrawal, all attempts will be made to follow up with the participant to establish the cause of withdrawal and to collect qualitative data regarding the experience of participation. All data, including those from withdrawn participants, will be included in the final analysis. If a participant withdraws from the study prematurely, we will consider replacing the participant if resources allow and if recruitment is ongoing for the study.

### Analysis plan

This study will generate rich longitudinal data across domains, including ADHD symptoms and impairment, co-occurring mental health problems, digital and cognitive markers, life transitions, and lifestyle behaviours, enabling a range of analyses. We will calculate descriptive statistics for participant demographics and RMT engagement, estimate attrition rates, and explore whether baseline characteristics predict drop-out or whether RMT data availability predicts subsequent outcome completion.

For Aim 1, we will examine how ADHD-related and contextual variables change and covary over time. We will visualise trajectories, estimate within- and between-person variance using generalised linear mixed models, and model individual-level change using spline-based random slope models. We will also examine how specific variable pairs covary over time—e.g., ADHD symptoms/impairment with co-occurring psychiatric symptoms, and with digital and cognitive markers. For each pair, we will estimate lagged associations using linear mixed models, distinguishing within- from between-person effects. This will allow us to identify dynamic patterns of change and factors that precede symptom fluctuations.

For Aim 2, we will develop multivariable prediction models for key outcomes identified in Aim 1, using regularised regression (elastic net) and tree-based machine learning (e.g. XGBoost). Model performance will be assessed using repeated, nested temporal cross-validation, with all modelling steps (e.g. pre-processing, imputation) repeated within each split to prevent data leakage. Models will include relevant baseline characteristics and participant-level covariates. To inform the development of a digital tool (Aim 3), we will build models that predict current-month outcomes based on predictors from preceding months (e.g. 1–3 months prior), incorporating lagged predictors and derived metrics such as changes in symptom severity or sleep variability.

### Data protection

Data acquired from the wearable devices and the RADAR-base Active and Passive Apps will be encrypted, pseudonymised, and uploaded automatically via Wi-Fi to secure, GDPR compliant servers managed by the RADAR-base and research team. Digital data from other sources, including limited identifiable data and pseudonymised data, will be stored securely on a GDPR-compliant, secure storage provider. Physical data, e.g. from cognitive ability tests, will be stored in local storage at King’s College London before being uploaded to a GDPR compliant, secure storage provider.

## Discussion

ART-transition will provide a unique set of active and passive remote monitoring data over 24 months from young people with ADHD as they transition to adulthood. The fine-grained data on fluctuations and changes in multiple outcome measures, and their wide-ranging potential predictors, will enable analyses in different timescales from hourly or daily changes for some measures, to monthly changes for others. No comparable prior dataset exists, with past ADHD follow-up studies incorporating limited data collection timepoints and measures.

Our extensive PPI work has indicated the need for new self-management tools for ADHD. People with lived experience highlighted the importance of technologies augmenting clinical services rather than replacing them, and noted that an app they could use personally would be helpful. Furthermore, evidence from other areas of adolescent development and mental health shows that adolescents use and want smartphone apps and websites to support healthy lifestyle knowledge and behaviours [60] and place more trust and value on evidence-based resources [61]. This study, therefore, allows us to identify real-world targets for intervention that can be integrated into a prototype of a smartphone app, which will be co-developed during the study. We will develop a prototype smartphone app for young transition-age people with ADHD. The app will aim to prevent negative outcomes and support healthy lifestyles in young people with ADHD by facilitating self-management, personalisation of treatment and engagement with adult services. Our approach will focus on giving young people with ADHD greater autonomy in how they manage their ADHD, in collaboration with their clinician. It will emphasise modifiable environmental factors and the prevention of negative outcomes. The unique data collection, ART system, RADAR-base platform, and our extensive co-design team – including young people with ADHD, the PPI panel, healthcare professionals involved in the clinical care of young people with ADHD, MindTech and The Hyve (a leading IT and data services provider in life sciences [62])– place us in a unique position to design a prototype for a new smartphone app.

A key challenge for RMT studies is missing data due to non-compliance from participants, such as participants not answering questionnaires on the Active App or not wearing the wearable device. To mitigate missing data, we have introduced regular automated app notifications and integrated feedback from our PPI focus groups (e.g. sending more notification reminders, sending notifications at the same time each day, and limiting the number of times participants are asked to complete cognitive tasks).

By using RMT in transition-age young people with ADHD to carry out unobtrusive, real-time data collection over a period of 24 months and by co-designing a prototype ADHD-transition digital tool, we will address the core questions of what changes take place during the transition to adulthood, what predicts such changes, and how can we prevent negative outcomes and support healthy lifestyles.

## Supplementary Information

Below is the link to the electronic supplementary material.

Supplementary Material 1

## Data Availability

No datasets were generated or analysed during the current study.

## Abbreviations

ADHD: Attention Deficit Hyperactivity Disorder
ART: ADHD Remote Technology
DSM-5: Diagnostic and Statistical Manual of Mental Disorders, Fifth Edition
RMT: Remote Measurement Technology
PPI: Patient and Public Involvement
CAMHS: Child and Adolescent Mental Health Services
