# Supplementary material for: ADHD remote technology and ADHD transition: predicting and preventing negative outcomes (ART-transition) - an adolescent prospective cohort study protocol
Source: BMC Psychiatry. 2025 Dec 11;25:1146. doi: 10.1186/s12888-025-07546-0 (PMC12696895; doi:10.1186/s12888-025-07546-0)
Supplement: Supplementary file 1 — Supplementary Material 1 [file 12888_2025_7546_MOESM1_ESM.docx]

**ADHD Remote Technology and ADHD Transition: Predicting and Preventing Negative Outcomes (ART-transition) - An Adolescent Prospective Cohort Study Protocol**

Supplemental Materials

Sample size calculations

Sample sizes were determined via a comprehensive simulation study and analytical methods. For Aim 1, we considered: (a) power to detect associations between pairs of repeatedly measured variables; (b) precision of the estimated within-person variance and intra-class correlation (ICC); and (c) the influence of missing data on these estimates. For Aim 2, we considered (d) the sample size required to develop multivariable prediction models.

We conducted a simulation study that replicated the planned procedures for data collection and analysis, simulating monthly assessments for a range of variables collected over two years. Means and standard deviations (SD) were derived from the ART-pilot study and past literature (See Table S1).

Table S1

| *Measure Mean (SD)* | *Mean (SD)* |
| --- | --- |
| *ADHD symptoms* | *33.0 (13.2)* |
| *ADHD impairment* | *17.8 (6.7)* |
| *Depression (PHQ-8)* | *9.7 (5.8)* |
| *Sleep stability* | *82.0 (31.0)* |
| *Physical activity (1)* | *44.5 (30.5)* |

Following past studies in adolescents with ADHD, we assumed an ICC of 0.30. All simulations were repeated 5000 times.

To estimate (a) the power to detect associations between pairs of variables (an outcome X_1_ and covariate X_2_), we fitted random intercept models under differing scenarios (varying sample and effect size). We considered effect sizes from small (standardised β = 0.1) to large (β = 0.8). Power was determined as the percentage of replications where the effect of interest was detected (p < 0.05). With 250 participants, we will be at least 80% powered to detect all but the smallest effect sizes. For example, we will have 90% power to detect effects of β = 0.2 or larger.

To estimate (b) the precision of the estimated variance, we fitted unconditional random intercept models with varying sample sizes and ICC. With 250 participants, we will be able to estimate an ICC for ADHD symptoms of 0.300 ± 0.04 (95% confidence limits of 0.256 and 0.342). Increasing the sample size beyond 250 did not substantially improve precision (e.g., 500 participants would give confidence intervals (CI) of ± 0.03).

To estimate (c), we considered two sources of missing data. First, we simulated data with varying lengths of follow-up (from 12-24 months). If individuals participate for 12 or 18 months, 95% CI of ICC for ADHD symptoms will be ± 0.05 or ± 0.04, respectively. Second, to consider the influence of missed assessments during follow-up, we simulated data with 0 to 50% of assessments missing at random and re-estimated precision and power. With 50% of assessments missing, power to detect a small effect (β = 0.2) was reduced but remained over 80% for most variables; precision for the ICC remained at ± 0.05. These numbers suggest the proposed sample size of 250 is sufficient for Aim 1, after allowing for missing data. It is also worth emphasising that past studies – RADAR-MDD (2) and ART-pilot (see p.6) – obtained highly encouraging retention and completion rates. RADAR-MDD reported 80% completion rates for primary outcome assessments across all follow-up timepoints. Of 623 participants enrolled, 80% participated for the maximum possible duration (enrolment to end of follow-up, up to 24 months).

To estimate (d), we calculated the minimum sample size required to develop a multivariable prediction model using the pmsampsize package for R (3). Previous RADAR studies using smartphone-based markers to predict clinical outcomes have reported R^2^ of 0.50 (4). To develop a model with 20 parameters for a continuous outcome (ADHD impairment; mean = 17.8; SD = 6.7; assuming shrinkage of 0.9) would require 250 observations given an R^2^ of 0.5. However, predictors and outcomes in the proposed study will be measured repeatedly over a 24-month period. If we conservatively assume that participants complete just 30% of assessments, this will still provide 1800 observations, exceeding the requirements for Aim 2.

References

1. Rommel AS, Lichtenstein P, Rydell M, Kuja-Halkola R, Asherson P, Kuntsi J, et al. Is Physical Activity Causally Associated With Symptoms of Attention-Deficit/Hyperactivity Disorder? J Am Acad Child Adolesc Psychiatry. 2015 Jul 1;54(7):565–70.

2. Simblett S, Matcham F, Siddi S, Bulgari V, Pietro CB di S, López JH, et al. Barriers to and Facilitators of Engagement With mHealth Technology for Remote Measurement and Management of Depression: Qualitative Analysis. JMIR MHealth UHealth. 2019 Jan 30;7(1):e11325.

3. Ensor J. pmsampsize: Sample Size for Development of a Prediction Model.

4. Zhang Y, Folarin AA, Sun S, Cummins N, Ranjan Y, Rashid Z, et al. Predicting Depressive Symptom Severity Through Individuals’ Nearby Bluetooth Device Count Data Collected by Mobile Phones: Preliminary Longitudinal Study. JMIR MHealth UHealth. 2021 Jul 30;9(7):e29840.
